# Mutation profile of non-small cell lung cancer revealed by next generation sequencing

**DOI:** 10.1186/s12931-020-01608-5

**Published:** 2021-01-06

**Authors:** Ya-Sian Chang, Siang-Jyun Tu, Yu-Chia Chen, Ting-Yuan Liu, Ya-Ting Lee, Ju-Chen Yen, Hsin-Yuan Fang, Jan-Gowth Chang

**Affiliations:** 1 Epigenome Research Center, China Medical University Hospital, 2 Yuh-Der Road, Taichung, 404 Taiwan; 2 Department of Laboratory Medicine, China Medical University Hospital, Taichung, Taiwan; 3 Center for Precision Medicine, China Medical University Hospital, Taichung, Taiwan; 4 Department of Medical Laboratory Science and Biotechnology, China Medical University, Taichung, Taiwan; 5 Department of Thoracic Surgery, China Medical University Hospital, Taichung, Taiwan; 6 School of Medicine, China Medical University, Taichung, Taiwan; 7 Department of Bioinformatics and Medical Engineering, Asia University, Taichung, Taiwan

**Keywords:** Non-small cell lung cancer, Whole-exome sequencing, Targeted gene sequencing, Trunk mutations

## Abstract

**Background:**

Precision therapy for lung cancer requires comprehensive genomic analyses. Specific effects of targeted therapies have been reported in Asia populations, including Taiwanese, but genomic studies have rarely been performed in these populations.

**Method:**

We enrolled 72 patients with non-small cell lung cancer, of whom 61 had adenocarcinoma, 10 had squamous cell carcinoma, and 1 had combined adenocarcinoma and squamous cell carcinoma. Whole-exome or targeted gene sequencing was performed. To identify trunk mutations, we performed whole-exome sequencing in two tumor regions in four patients.

**Results:**

Nineteen known driver mutations in *EGFR*, *PIK3CA*, *KRAS*, *CTNNB1*, and *MET* were identified in 34 of the 72 tumors evaluated (47.22%). A comparison with the Cancer Genome Atlas dataset showed that *EGFR* was mutated at a much higher frequency in our cohort than in Caucasians, whereas *KRAS* and *TP53* mutations were found in only 5.56% and 25% of our Taiwanese patients, respectively. We also identified new mutations in *ARID1A*, *ARID2*, *CDK12*, *CHEK2*, *GNAS*, *H3F3A*, *KDM6A*, *KMT2C*, *NOTCH1*, *RB1*, *RBM10*, *RUNX1*, *SETD2*, *SF3B1*, *SMARCA4*, *THRAP3*, *TP53*, and *ZMYM2*. Moreover, all ClinVar pathogenic variants were trunk mutations present in two regions of a tumor. RNA sequencing revealed that the trunk or branch genes were expressed at similar levels among different tumor regions.

**Conclusions:**

We identified novel variants potentially associated with lung cancer tumorigenesis. The specific mutation pattern in Taiwanese patients with non-small cell lung cancer may influence targeted therapies.

## Background

Lung cancer is the leading cause of cancer-associated mortality worldwide. An estimated 2.09 million new lung cancer cases and 1.76 million lung cancer-associated mortalities were reported in the GLOBOCAN 2018 database [1]. Lung cancer can be divided into two broad categories according to histology: small-cell lung cancer and non-small-cell lung cancer (NSCLC). The latter comprises more than 80–85% of all lung cancers. NSCLC is subdivided into adenocarcinoma (ADC; 60%), squamous cell carcinoma (SqCC; 30–35%), large cell carcinoma, and other rare tumors, including adenosquamous carcinoma [2]. Risk factors include tobacco consumption, genetic susceptibility, poor diet, air pollution and occupational exposures, such as asbestos, metals and mixed occupation exposures, silica, polycyclic aromatic hydrocarbons, and diesel exhaust fumes [3]. Tobacco smoking is the main risk factor for lung cancer; however, 10–15% of those diagnosed with lung cancer are never-smokers [4]. Epidemiologic studies reveal that the proportion of lung cancer in never-smokers is higher in East Asia [5]. Never-smoker East Asian females are diagnosed more often with ADC, and these patients exhibit higher treatment response rates to epidermal growth factor receptor (EGFR) tyrosine kinase inhibitors [6]. Chest radiography, sputum cytology, and low-dose computed tomography (CT) have been used for lung cancer screening. Despite advances in genomic research and targeted therapies, leading to improvements in therapeutic strategies and the clinical outcomes of lung cancer patients [7], the overall 5-year survival rate of lung cancer remains very low (16.8%) [8]. The prognosis of lung cancer remains poor because most patients are often diagnosed at an advanced stage.

Rapid advancements in next-generation sequencing technology and a better understanding of cancer biology have provided unprecedented opportunities to characterize the genome of human tumors including lung cancer. The Cancer Genome Atlas (TCGA) lung cancer working group has profiled and analyzed 230 ADC and 178 SqCC specimens to identify molecular aberrations at the DNA, RNA, protein, and epigenetic levels [9, 10]. In ADC, the most common mutations are *TP53*, *KRAS*, *EGFR*, *NF1*, *BRAF*, *MET*, and *RIT*. Pathway alterations in ADC are involved in RTK/RAS/RAF, mTOR, JAK-STAT, DNA repair, cell regulation, and epigenetic deregulation. Mutations in *TP53*, *CDKN2A*, *PIK3CA*, *NFE2L2*, *KEAP1*, *CUL3*, *PTEN*, *NF1*, *NOTCH1*, *2* and *3*, *DDR2*, and *EGFR* genes are frequently observed in SqCC. Pathway alterations in SqCC involve squamous differentiation, the oxidative stress response, PIK3CA, DNA repair, cell cycle regulation, and epigenetic deregulation [11]. ADC and SqCC show genetic alterations or gene expression differences [12, 13]. In ADC, the most common therapeutic targets are *EGFR* and *BRAF* mutations and *ALK* and *ROS1* rearrangements. Molecular genotyping is now routine in ADC. In SqCC, targeted agents are largely ineffective, and many targeted therapies are currently undergoing clinical trials [14].

To develop a more comprehensive genomic picture of NSCLC, we performed whole-exome sequencing (WES) or targeted gene sequencing (TGS) in 72 Taiwanese patients with NSCLC. In addition, we compared the results with the TCGA NSCLC dataset, which involves mainly Western populations. We also investigated the associations between genetic alterations and clinicopathological features.

## Materials and methods

### Subjects and DNA extraction

A total of 61 fresh-frozen and 11 formalin-fixed paraffin-embedded (FFPE) specimens were obtained from 72 Taiwanese patients with lung cancer who underwent surgical resection from May 2007 to April 2019 at the China Medical University Hospital. The 72 lung tumors comprised 61 ADCs, 10 SqCCs, and 1 combined ADC and SqCC. DNA from frozen tissues and FFPE specimens was extracted using the QIAamp® DNA Micro Kit (Qiagen, Heidelberg, Germany) and QIAamp® DNA FFPE Tissue Kit (Qiagen) according to the manufacturer’s instructions. The DNA concentration was quantified using the NanoDrop1000 spectrophotometer (Nanodrop Technologies, Wilmington, DE, USA) and a Qubit Fluorometer (Invitrogen, Carlsbad, CA, USA).

### TGS and data analysis

TGS was performed using the Qiagen platform with a panel that included either 275 (cat. no. DHS-3501Z) or 72 genes (cat. no. DHS-005Z). DNA libraries were prepared using components from the QIAseq Targeted DNA Panel Kit (Qiagen) and QIAseq Targeted DNA Panel Human Lung Cancer Panel (Qiagen). Briefly, 80 ng DNA was enzymatically fragmented and end-repaired in a 25-μl reaction volume containing 2.5 μl 10 × fragmentation buffer and 5 μl fragmentation enzyme mix. The reaction was carried out at 4 °C for 1 min, 32 °C for 24 min, and 65 °C for 30 min. Next, 10 μl 5 × ligation buffer, 5 μl DNA ligase, and 2.8 μl 25 μM barcoded adapters were added along with enough water to reach a reaction volume of 50 μl. Reaction tubes were then incubated at 20 °C for 15 min. To ensure complete removal of free barcoded adapters, each reaction was purified using 1.4 × (or 1.0 ×) QIAseq beads for two rounds. The purified DNA was then mixed in a 20-μl reaction volume with 10 nM each target primer, 400 nM IL-Forward primer, 1 × TEPCR buffer, and 0.8 μl HotStarTaq DNA polymerase. The PCR protocol was as follows: 95 °C for 13 min; 98 °C for 2 min; six cycles of 98 °C for 15 s and 65 °C for 15 min; and 72 °C for 5 min. Each reaction was cleaned once using 1.4 × (or 1.0 ×) QIAseq beads to remove unused primers. Enriched DNA was combined with 400 nM IL-Index primers, 1 × UPCR buffer, and 1 μl HotStarTaq DNA polymerase in a volume of 20 μl. The universal PCR conditions were as follows: 95 °C for 13 min; 98 °C for 2 min; 20 cycles of 98 °C for 15 s and 60 °C for 2 min; and 72 °C for 5 min. The DNA library was purified once using 1.4 × (or 1.0 ×) QIAseq beads and quantified using Qubit Fluorometric Quantitation (Thermon Fisher Scientific-US, Waltham, MA, USA). Libraries were sequenced on Illumina NextSeq (paired-end, 2 × 150 bp) according to the manufacturer’s instructions (Illumina, San Diego, CA, USA). TGS analysis was described in detail in our previous work [15].

### WES and data analysis

A total of 50 ng DNA (based on Qubit quantification) was tagmented by a transposome, followed by clean-up and amplification of the tagmented DNA. A 200–400 bp band was selected, and exome capture was performed using the Nextera Exome Library Preparation Kit (Illumina). The DNA library was quantified using the Qubit 3.0 Fluorometer (Invitrogen) and Agilent 4200 Bioanalyzer (Agilent Technologies, Santa Clara, CA, USA). Samples were subjected to paired-end sequencing using the Illumina NovaSeq 6000 platform with a 150-bp read length. WES analysis has been described in detail in our previous work [16].

### RNA sequencing (RNA-seq)

Total RNA was extracted from clinical tissue samples using the NucleoSpin**®** RNA Kit (MACHEREY–NAGEL GmgH, Düren*,* Germany) following the manufacturer’s instructions. The quality, quantity, and integrity of the total RNA were evaluated using the NanoDrop1000 spectrophotometer and Bioanalyzer 2100 (Agilent Technologies). Samples with an RNA integrity number > 6.0 were used for RNA-seq. An mRNA-focused, barcoded library was generated using the TruSeq strand mRNA Library Preparation Kit (Illumina). The libraries were sequenced on the Illumina Nova Seq 6000 instrument (Illumina), using 2 × 151-bp paired-end sequencing flow cells following the manufacturer’s instructions.

### RNA-seq data analysis

Illumina bcl2fastq Conversion Software (v2.20.0.422) was utilized to convert raw sequencing data to fastq format (Illumina). Trimmomatic PE (v0.39) was applied to control the read quality and remove sequencing adapters [17]. Reads were discarded if their average quality was < 20 (AVGQUAL:20) and their read length < 105 bp (MINLEN:105). Next, paired quality-controlled reads were aligned to the human genome (GRCh38), and gene expression was quantified using transcripts per million normalization via the HISAT2 (2.1.0) [18] and StringTie (1.3.5) [19] pipelines. To evaluate the similarities between different regions from the same tissue, we applied Spearman’s rank correlation of the transcripts per million values of 299 gene signatures [20]. A heatmap of the gene expression values was plotted using Morpheus (https://software.broadinstitute.org/morpheus), and the correlation coefficients were visualized using Seaborn, a Python data visualization library (https://github.com/mwaskom/seaborn).

### Statistical analysis

All statistical analyses were performed using SPSS software ver. 22.0. Chi-squared or Fisher’s exact tests were used to compare two categorical variables. Survival analysis was performed using Kaplan–Meier survival plot and log-rank test. A p-value less than 0.05 was considered statistically significant.

## Results

### Patient characteristics

The patients in this study comprised 39 males and 33 females, with a mean age of 62.35 years, of whom 61 had ADC, 10 had SqCC, and 1 had combined ADC and SqCC. Stage I disease was identified in 38 patients, stage II in 12 patients, stage III in 10 patients, and stage IV in 6 patients (Table 1).

**Table 1:** Description of Taiwanese NSCLC cases

Variable	No of patients N = 72 (%)
Age (years)	
Mean ± SD	62.35 ± 13.59
Range	36–83
Gender	
Male	39
Female	33
Clinical stage	
I	38 (55.78)
II	12 (16.67)
III	10 (13.89)
IV	6 (8.33)
Missing	6 (8.33)
Histology type	
Adenocarcinoma	61 (84.72)
Squamous cell carcinoma	10 (13.89)
ADC + SqCC	1 (1.39)
Smoking status	
Non-smokers	47 (65.28)
Smokers	25 (34.72)

### Genomic alterations

Among the 72 samples, 32 contained driver mutations in well-known cancer genes in NSCLC, such as *EGFR* (n = 26; E709G, T790M, L858R and non-frameshift deletions of exon 19), and *PIK3CA* (n = 4; E542K and G1049R). Besides *EGFR* and *PIK3CA*, other known mutations were detected in *KRAS* (n = 4; G12V, G12A, G12D, and Q61H), which have all been reported as driver mutations in lung cancer. In addition, four samples carried known activating mutations in the well-known oncogenes *CTNNB1* (n = 3; S33F, S37C and S37F) and *MET* (n = 1; R1004X and c.3028 + 1G > T). Overall, 34 specimens harbored driver mutations in five cancer genes (*EGFR*, *PIK3CA*, *KRAS*, *CTNNB1*, and *MET*), which are canonical driver mutations (Additional file 1: Table S1). These mutations were mutually exclusive, except for four cases of double mutations (n = 2; *EGFR* and *CTNNB1* and n = 2; *EFGR* and *PIK3CA*). *TP53* was the most frequently mutated gene after *EGFR* (n = 18; S95fs, K120X, T125T, W146X, 152_153del, V173A, F212fs, G245C, G245D, R248L, R248W, R273H, R273C, V274D, E286K, and c.673-1G > T) (Additional file 1: Table S1). Among the 16 *TP53* variants, two were novel (S95fs and F212fs).

### Comparisons between Taiwanese and Caucasian patients with NSCLC

**Fig. 1:**
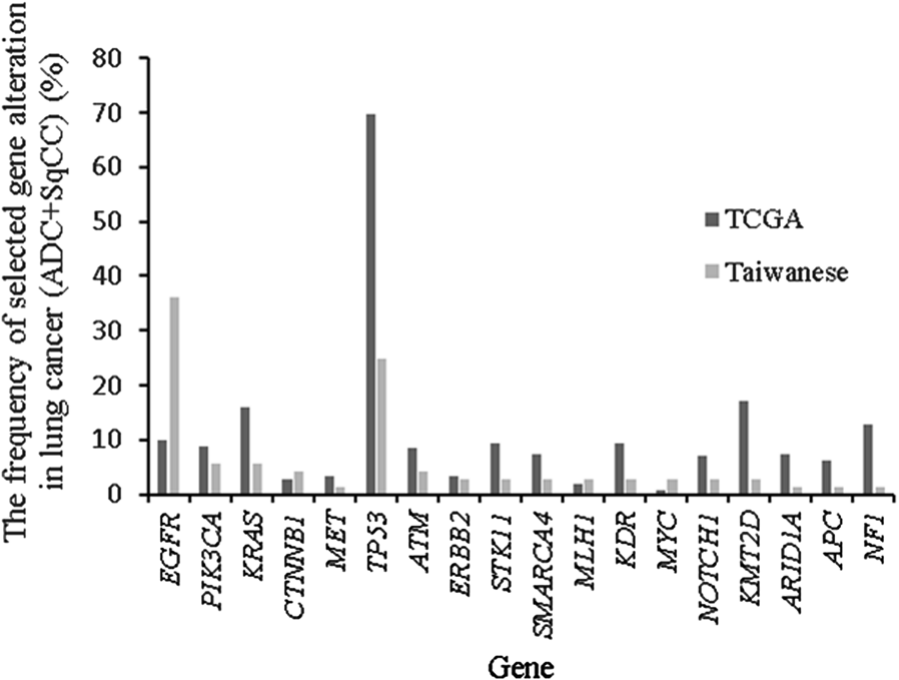
Comparison of the frequencies of selected gene alterations between the Taiwaneses and TCGA cohorts

To compare the frequency of driver mutations of NSCLC between Taiwanese and Caucasian patients, we obtained all available lung cancer cases (560 ADC and 489 SqCC) from the TCGA dataset. Notable differences from the TCGA data included the frequencues of mutations in *EGFR* (36.11% vs. 9.82%, p < 0.0001), *KRAS* (5.56% vs. 15.92%, p = 0.0165), and *TP53* (25.00% vs. 69.69%, p < 0.0001). A full comparison of the frequencies of selected gene alterations between the two cohorts is depicted in Fig. 1 and Additional file 1: Table S2.

### Clinically relevant genomic alterations

Based on the latest NSCLC guidelines published by the National Comprehensive Cancer Network, clinically relevant genomic alterations were identified in 34 (47.22%) patients (Table 2). As shown in Table 2, the clinically relevant alterations included in *EGFR* (26, 36.11%), *ERBB2* (2, 2.78%), *KRAS* (4, 5.56%), *MET* (1, 1.38%), and *NTRK1* (1, 1.38%).

**Table 2:** Genomic alterations associated with targeted therapies

Gene	Alteration	Targeted therapy	Frequency
Any gene (s)			34
*EGFR*	L858R + E709G	Gefitinib	1
	Exon 19 deletion	Erlotinib, Gefitinib, Afatinib	12
	L858R + T790M	Osimertinib	1
	L858R	Erlotinib, Gefitinib, Afatinib	12
*KRAS*	G12V	Resistance to Erlotinib and Gefitinib	1
	G12A	Resistance to Erlotinib and Gefitinib	1
	G12D	Resistance to Erlotinib and Gefitinib	1
	Q61H	Resistance to Erlotinib and Gefitinib	1
*ERBB2*	Insertion	Afatinib, Dacomitinib, Trastuzumab	2
*MET*	R1004X	Crizotinib	1
	c.1738 + 1G > T		
*NTRK1*	R646C	Entrectinib	1

Among the 26 patients with *EGFR* mutation patients, only 8 had an additional *TP53* mutation, of whom 1 died of a cause unrelated to NSCLC. Among the 7 remaining patients, 3 had good survival outcomes, and 4 did not. We compared the genetic differences between the patients with a good and those with a poor survival outcome. In addition to the *EGFR* and *TP53* mutations, one patient with poor survival harbored a *MYC* non-frameshift deletion (p.48_48del, rs776629119), and one patient with good survival had an *AR* non-frameshift insertion (p.L57delinsLQQQ, rs4045402) and a *FBXW7* non-frameshift deletion (p.117_117del, rs781154022), and another patient with good survival had a *CTNNB1* (p.S37F, rs121913403) mutation.

### Correlations between driver mutations and clinicopathological characteristics

Correlations between the genotypes and clinicopathological characteristics are listed in Table 3. The *EGFR* mutation rate was significantly higher in patients with ADC than in those with SqCC (41.0% vs. 10.0%, p = 0.059). Moreover, the *EGFR* mutation rate was significantly higher in patients without smoking than in those with smoking (44.7% vs. 20.0%, p = 0.038). No association was found between the *EGFR* mutation status and sex, age, or tumor stage of the patients. In contrast, the *PIK3CA* and *TP53* mutation rates were significantly higher in patients with SqCC than in those with ADC (30.0% vs. 1.6%, p = 0.008 and 50.0% vs. 21.3%, p = 0.053). Furthermore, the *TP53* mutation rate was significantly higher in patients with smoking than in those without smoking (40.0% vs. 17.0%, p = 0.032). No association was found between *KRAS* or *CTNNB1* mutations and any clinicopathological characteristic.

**Table 3:** Correlation of *EGFR*, *PIK3CA*, *KRAS*, *TP53*, and *CTNNB1* mutations with clinicopathological features

Features		*EGFR* mutation			p-value	*PIK3CA* mutation			p-value	*KRAS* mutation			p-value	*TP53* mutation			p-value	*CTNNB1* mutation			p-value
		No	Yes	Total		No	Yes	Total		No	Yes	Total		No	Yes	Total		No	Yes	Total	
Gender	Male	27	12	39	0.305	38	1	39	0.327	36	3	39	0.620	27	12	39	0.219	39	0	39	0.091
	Female	19	14	33		30	3	33		32	1	33		27	6	33		30	3	33	
Age	< 62	24	12	36	0.624	33	3	36	0.303	33	3	36	0.303	25	11	36	0.276	35	1	36	1.000
	≥ 62	22	14	36		35	1	36		35	1	36		29	7	36		34	2	36	
Stage	I–II	30	20	50	0.859	48	2	50	1.000	47	3	50	1.000	36	14	50	0.461	48	2	50	1.000
	III–IV	10	6	16		15	1	16		15	1	16		13	3	16		15	1	16	
Histology type	ADC	36	25	61	**0.059**	60	1	61	**0.008**	57	4	61	1.000	48	13	61	**0.053**	58	3	61	1.000
	SqCC	9	1	10		7	3	10		10	0	10		5	5	10		10	0	10	
Smoking status	Non-smokers	26	21	47	**0.038**	44	3	47	1.000	46	1	47	0.117	39	8	47	**0.032**	44	3	47	0.547
	Smokers	20	5	25		24	1	25		22	3	25		15	10	25		25	0	25	

*p* value by Chi-square or Fisher’s exact test when appropriated

Bold values represent* p* < 0.05 or borderline

**Fig. 2:**
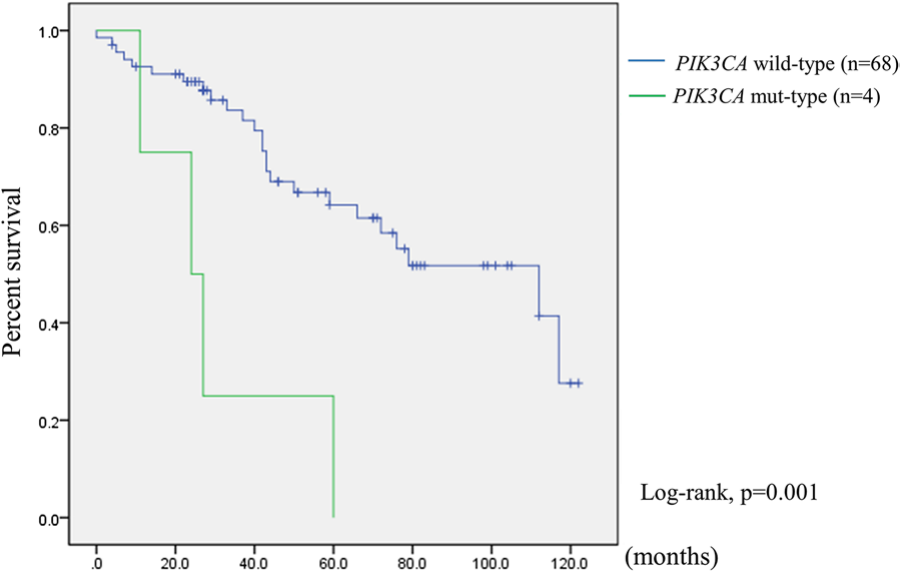
Kaplan–Meier survival curve of patients with *PIK3CA* mutations

We used Kaplan–Meier curve analysis to assess overall survival. In our cohort, *PIK3CA* mutation was a prognostic of worse overall survival (Fig. 2). There was, however, no significant difference in mortality between *EGFR*, *KRAS*, *TP53*, and *CTNNB1* mutations (Additional file 2: Figure S1).

### Identification of trunk or branch driver mutations

**Fig. 3:**
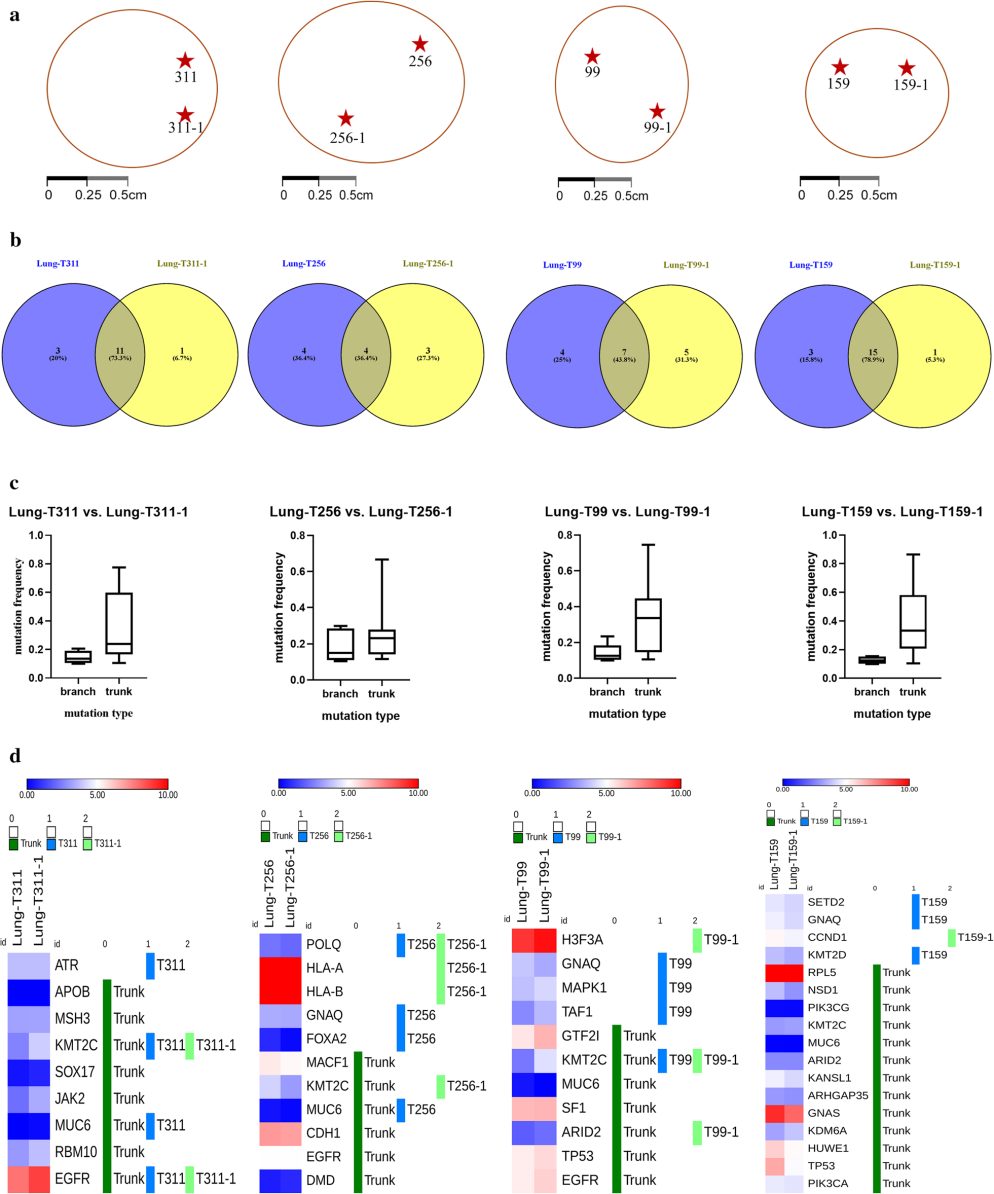
Intratumoral genetic heterogeneity and homogeneity in four patients with NSCLC. **a** The regions harvested from the same surgically resected NSCLC. **b** Distribution of trunk and branch mutations in each region of the samples. **c** Mutation frequencies of trunk and branch mutations. **d** Gene expression analysis of the trunk and branch genes

To exame intratumor heterogeneity, we applied multi-region WES in 8 tumor regions from 4 resected tissues (Fig. 3a). In order to determine whether driver genes carry trunk or branch mutations, we identified potential driver mutations among the 299 known cancer driver genes [20]. All variants classified as pathogenic in the ClinVar database are trunk mutations present in two tumor regions (Additional file 1: Table S3). Among the 61 predicted pathogenic variants identified from four patients (15, 11, 16, and 19, respectively), 37 were classified as trunk mutations (11, 4, 7, and 15, respectively) (Fig. 3b, Additional file 1: Table S4).

We further analyzed the variant allele frequencies of the trunk and branch mutations. Generally, the variant allele frequencies in four paired samples suggested that trunk mutations (median: 0.23–0.34%) occurred much more frequently than branch mutations (median: 0.12–0.15%) (Fig. 3c).

We also analyzed the expression of the driver genes that carried trunk or branch mutations. Gene expression profiles revealed no differences in driver genes harboring trunk or branch mutations between the two different tumor regions of the four paired samples (Fig. 3d).

### Intratumoral heterogeneity of 299 driver genes

**Fig. 4:**
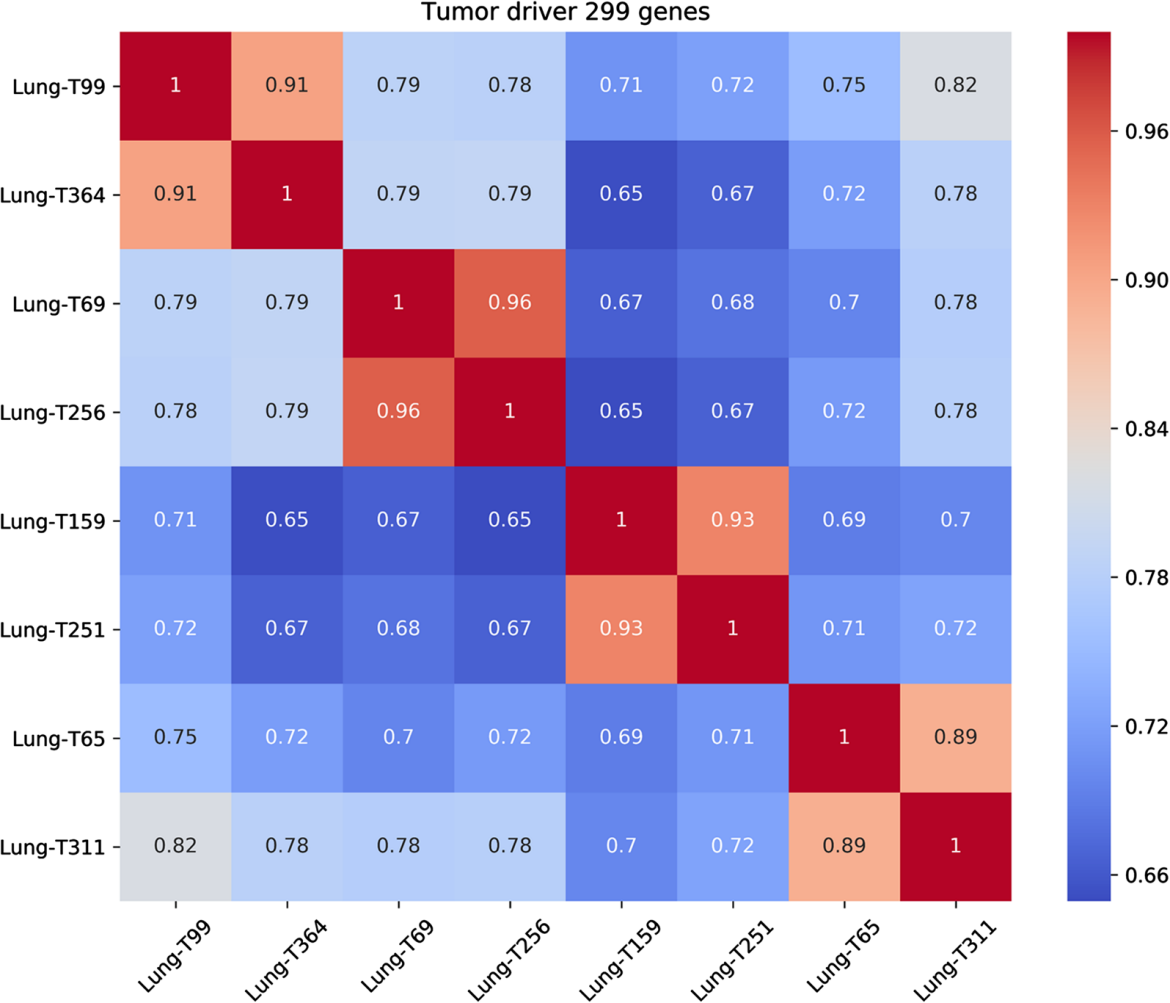
Spearman rank correlations assessing the consistency in gene expression between two samples from four tumors each.

We determined the intratumoral expression of 299 driver genes, which were derived from 33 cancer types in the PanCancer dataset [20]. We used Spearman’s rank correlation to calculate the gene expression correlations between two regions from four tumors each. Two regions from a tumor showed the highest correlation coefficient (Fig. 4).

The numbers of differentially expressed genes with a fold change in expression > 4 in the four paired samples were 4 (*FGFR2*, *PRKAR1A*, *MYC*, and *MYD88*), 1 (*MYD88*), 4 (*KMT2C*, *GNA11*, *ALB*, and *B2M*), and 2 (*KLF5* and *CDKN2A*), respectively (Additional file 1: Table S5). Most of the genes showed consistent expression (fold change ≤ 4). There were few differences between the different regions within the same tumor, and we suggest that any differences were due to branch mutations. Thus, the 299-driver gene signature may correctly predict cancer etiology if assessed from a single tumor region.

## Discussion

In the present study, we successfully performed comprehensive genomic profiling in tumor specimens from 72 Taiwanese NSCLC patients using WES or TGS. We found that *EGFR* mutations were more common in patients with ADC, irrespective of sex, age, or tumor stage. *PIK3CA* and *TP53* mutation rates were also higher in patients with SqCC. A comparison of driver gene mutations in our NSCLC patients with the TCGA dataset showed that *EGFR* was mutated at a much higher frequency in our Taiwanese cohort compared with Caucasians. In contrast, *KRAS*, *BRAF*, and *TP53*, the most common mutated genes in Caucasians, were found in only 5.56, 0, and 25%, respectively, of the Taiwanese NSCLC patients in our study, which is consistent with other studies [12, 21]. These differences are most likely due to racial and environmental differences.

In addition, we also identified 83 novel variants in 72 genes. Eighteen of these have been reported as cancer driver genes (*ARID1A*, *ARID2*, *CDK12*, *CHEK2*, *GNAS*, *H3F3A*, *KDM6A*, *KMT2C*, *NOTCH1*, *RB1*, *RBM10*, *RYNX1*, *SETD2*, *SF3B1*, *SMARCA4*, *THRAP3*, *TP53* and *ZMYM2*) (Additional file 1: Table S6) [20]. Eight driver genes were associated with LUAD and LUSC.

*ARID1A* encodes a member of the SWI/SNF family of proteins. Mutation of *ARID1A* has been documented in a number of cancers [22], and approximately 8% of lung ADCs contain mutations in *ARID1A* [23]. In this study, we identified a novel *ARID1A* mutation (p.G933fs) in a patient with SqCC.

*KDM6A* is located on chromosome Xp11 and encodes a tetratricopeptide repeat protein. The protein contains a Jumonji C domain and catalyzes the demethylation of tri/dimethylated histone H3. *KDM6A* is a tumor suppressor gene in different cancers, including lung SqCC [20]. In this study, we identified a novel *KDM6A* mutation (p.S314X) in a patient with SqCC.

NOTCH1, a member of the NOTCH protein family, contains an extracellular domain consisting of multiple epidermal growth factor-like repeats (36) and an intracellular domain consisting of multiple domain types. *NOTCH1* is reported to have a bimodal role as a tumor suppressor and an oncogene in several cancers [24, 25]. In this study, we identified a novel *NOTCH1* mutation (p.E2534K) in a patient with SqCC.

*RB1* encodes a negative regulator of the cell cycle protein and was the first tumor suppressor gene identified. Its tumor suppressive function is due to inhibition of the transcription factor E2F1. Any defect in the *RB1* gene causes cells to transition from the G1 to S-phase of the cell cycle [26]. *RB1* is inactivated in a wide range of cancers, including lung ADC and SqCC. In this study, we identified a novel *RB1* mutation (p.I848fs) in a patient with ADC. The I848fs mutation occurs in the C domain, which mediates the interaction with E4F1 [27].

*RBM10*, a tumor suppressor gene [28, 29], encodes a nuclear protein that contains an RNA-recognition motif. RBM10 regulates pre-mRNA splicing in the alternative splicing pathway [30]. In this study, we identified a novel *RBM10* mutation (p.A410fs) in a patient with ADC.

*SETD2* encodes a protein that interacts with huntingtin. The protein is a histone methyltransferase responsible for the tri-methylation of lysine 36 on histone H3 (H3K36me3), using H3K36me2 as a substrate [31]. *SETD2* is a tumor suppressor gene expressed in different cancer types [32]. In this study, we identified a novel *SETD2* mutation (p.L1525P), present in the AWS domain, in a patient with SqCC.

*SMARCA4*, also known as *BRG1*, encodes a member of the SWI/SNF family, which possesses helicase and ATPase activities. Human SWI/SNF enzyme subunits are mutated in approximately 20% of cancers [33]. *SMARCA4* is possibly tumor supressive in lung ADC. In contrast, *SMARCA4* may also be an oncogene in liver hepatocellular carcinoma, lower-grade glioma, and pan-cancer [20]. In this study, we identified a novel *SMARCA4* mutation (p.Q570fs) in a patient with ADC.

Recently, Skoulidis et al. reported that co-occurring genomic alterations affect the response of NSCLC to anticancer therapies [34]. The mean overall survival was 82.30 ± 9.80 months for the *EGFR* mutant *TP53* wildtype cohort, 86.88 ± 15.41 months for the *EGFR/TP53*-mutant cohort; p = 0.839 (Additional file 2: Figure S2). Among them, 11 patients used EGFR-TKI therapy. We also assessed whether there are differences in survival after EGFR-TKI therapy. The mean overall survival was 58.29 ± 10.26 months for the *EGFR* mutant *TP53* wildtype cohort (n = 8), 114.50 ± 2.50 months for the *EGFR/TP53*-mutant cohort (n = 3); p = 0.216 (Additional file 2: Figure S3). The difference in survival time between these two group was not significant, which may due to small sample size. We also assessed the overall survival of patients with *EGFR*/*TP53*-mutant lung cancers. Our cohort contained eight patients with co-existing *EGFR*/*TP53*-alterations. We compared the mutational signatures between the patients with a good and those with a poor survival outcome. The presence of a co-existing *MYC* alteration was associated with worse survival in patients with *EGFR*/*TP53*-mutant lung ADCs. Moreover, a co-existing *AR*, *FBXW7*, or *CTNNB1* alteration was associated with better survival in patients with *EGFR*/*TP53*-mutant lung ADCs. In our study, a relatively small number of patients with co-existing *EGFR*/*TP53* alterations was identified.

Intratumoral heterogeneity presents a major challenge in precision cancer therapy because it can lead to underestimation of the tumor genomic landscape when based on single tumor biopsy samples, and this might contribute to drug resistance and treatment failure [35]. All known ClinVar pathogenic mutations were identified in all regions of individual tumors. The predicted pathogenic variants were trunk mutations, with a frequency ranging from 36.36 to 78.95%. We found that the trunk or branch mutations were expressed at a constant level based on the transcriptome data. We found few genes with varying expression levels in different regions of the same sample, and our results differ slightly from those of other study [36], which may be because our selected regions were close in proximity.

In summary, we identified genomic aberrations underlying NSCLC in a Taiwanese population. Our study provides putative biomarkers for prognostic prediction in lung cancer. Further research is required to elucidate the functions of these genes and their pathways.

## Supplementary Information

**Additional file 1: Table S1.** Mutations in five cancer genes in the 72 patients. **Table S2.** Comparison of driver gene mutations of lung cancer between Taiwanese patients and the Caucasian cohort in TCGA dataset. **Table S3.** List of trunk mutations of ClinVar pathogenic variants in four paired samples. **Table S4.** List of trunk and branch mutations of prediction pathogenic variants in four paired samples. **Table S5.** List of differentially expressed genes with a fold change in expression > 4 in four paired samples. **Table S6.** Novel mutations in cancer driver genes.

**Additional file 2: Figure S1.** Kaplan–Meier survival curve of patients with *EGFR*, *KRAS*, *TP53*, and *CTNNB1* mutations. **Figure S2.** Kaplan–Meier survival curve of 18 patients with *EGFR* mutation *TP53* wild-type versus 8 patients with *EGFR/TP53* mutations. **Figure S3.** Kaplan–Meier survival curve of 8 patients with *EGFR* mutation *TP53* wild-type versus 3 patients with *EGFR/TP53* mutations after EGFR-TKI treatment.

## References

[1] Ferlay J, Colombet M, Soerjomataram I, Mathers C, Parkin DM, Pineros M, Znaor A, Bray F (2019). Estimating the global cancer incidence and mortality in 2018: GLOBOCAN sources and methods. Int J Cancer.

[2] Siegel R, Ma J, Zou Z, Jemal A (2014). Cancer statistics, 2014. CA Cancer J Clin.

[3] Malhotra J, Malvezzi M, Negri E, La Vecchia C, Boffetta P (2016). Risk factors for lung cancer worldwide. Eur Respir J.

[4] Belani CP, Marts S, Schiller J, Socinski MA (2007). Women and lung cancer: epidemiology, tumor biology, and emerging trends in clinical research. Lung Cancer.

[5] Zhou F, Zhou C (2018). Lung cancer in never smokers-the East Asian experience. Transl Lung Cancer Res.

[6] Ha SY, Choi SJ, Cho JH, Choi HJ, Lee J, Jung K, Irwin D, Liu X, Lira ME, Mao M (2015). Lung cancer in never-smoker Asian females is driven by oncogenic mutations, most often involving EGFR. Oncotarget.

[7] Hirsch FR, Scagliotti GV, Mulshine JL, Kwon R, Curran WJ, Wu YL, Paz-Ares L (2017). Lung cancer: current therapies and new targeted treatments. Lancet.

[8] Kanodra NM, Silvestri GA, Tanner NT (2015). Screening and early detection efforts in lung cancer. Cancer.

[9] Cancer Genome Atlas Research N (2014). Comprehensive molecular profiling of lung adenocarcinoma. Nature.

[10] Cancer Genome Atlas Research N (2012). Comprehensive genomic characterization of squamous cell lung cancers. Nature.

[11] Devarakonda S, Morgensztern D, Govindan R (2015). Genomic alterations in lung adenocarcinoma. Lancet Oncol.

[12] Zhang XC, Wang J, Shao GG, Wang Q, Qu X, Wang B, Moy C, Fan Y, Albertyn Z, Huang X (2019). Comprehensive genomic and immunological characterization of Chinese non-small cell lung cancer patients. Nat Commun.

[13] Faruki H, Mayhew GM, Serody JS, Hayes DN, Perou CM, Lai-Goldman M (2017). Lung adenocarcinoma and squamous cell carcinoma gene expression subtypes demonstrate significant differences in tumor immune landscape. J Thorac Oncol.

[14] Friedlaender A, Banna G, Malapelle U, Pisapia P, Addeo A (2019). Next generation sequencing and genetic alterations in squamous cell lung carcinoma: where are we today?. Front Oncol.

[15] Chang YS, Fang HY, Hung YC, Ke TW, Chang CM, Liu TY, Chen YC, Chao DS, Huang HY, Chang JG (2018). Correlation of genomic alterations between tumor tissue and circulating tumor DNA by next-generation sequencing. J Cancer Res Clin Oncol.

[16] Chang CC, Chang YS, Huang HY, Yeh KT, Liu TC, Chang JG (2018). Determination of the mutational landscape in Taiwanese patients with papillary thyroid cancer by whole-exome sequencing. Hum Pathol.

[17] Bolger AM, Lohse M, Usadel B (2014). Trimmomatic: a flexible trimmer for Illumina sequence data. Bioinformatics.

[18] Kim D, Paggi JM, Park C, Bennett C, Salzberg SL (2019). Graph-based genome alignment and genotyping with HISAT2 and HISAT-genotype. Nat Biotechnol.

[19] Pertea M, Pertea GM, Antonescu CM, Chang TC, Mendell JT, Salzberg SL (2015). StringTie enables improved reconstruction of a transcriptome from RNA-seq reads. Nat Biotechnol.

[20] Bailey MH, Tokheim C, Porta-Pardo E, Sengupta S, Bertrand D, Weerasinghe A, Colaprico A, Wendl MC, Kim J, Reardon B (2018). Comprehensive characterization of cancer driver genes and mutations. Cell.

[21] Liu L, Liu J, Shao D, Deng Q, Tang H, Liu Z, Chen X, Guo F, Lin Y, Mao M (2017). Comprehensive genomic profiling of lung cancer using a validated panel to explore therapeutic targets in East Asian patients. Cancer Sci.

[22] Takeda T, Banno K, Okawa R, Yanokura M, Iijima M, Irie-Kunitomi H, Nakamura K, Iida M, Adachi M, Umene K (2016). ARID1A gene mutation in ovarian and endometrial cancers (review). Oncol Rep.

[23] Imielinski M, Berger AH, Hammerman PS, Hernandez B, Pugh TJ, Hodis E, Cho J, Suh J, Capelletti M, Sivachenko A (2012). Mapping the hallmarks of lung adenocarcinoma with massively parallel sequencing. Cell.

[24] Westhoff B, Colaluca IN, D'Ario G, Donzelli M, Tosoni D, Volorio S, Pelosi G, Spaggiari L, Mazzarol G, Viale G (2009). Alterations of the Notch pathway in lung cancer. Proc Natl Acad Sci USA.

[25] Fukusumi T, Califano JA (2018). The NOTCH pathway in head and neck squamous cell carcinoma. J Dent Res.

[26] Burkhart DL, Sage J (2008). Cellular mechanisms of tumour suppression by the retinoblastoma gene. Nat Rev Cancer.

[27] Fajas L, Paul C, Zugasti O, Le Cam L, Polanowska J, Fabbrizio E, Medema R, Vignais ML, Sardet C (2000). pRB binds to and modulates the transrepressing activity of the E1A-regulated transcription factor p120E4F. Proc Natl Acad Sci USA.

[28] Hernandez J, Bechara E, Schlesinger D, Delgado J, Serrano L, Valcarcel J (2016). Tumor suppressor properties of the splicing regulatory factor RBM10. RNA Biol.

[29] Ji Y, Xie S, Jiang L, Liu L, Li L, Luo L, Chen Y, Zhang J, Yu L, Zhang Y (2017). Increased cell apoptosis in human lung adenocarcinoma and in vivo tumor growth inhibition by RBM10, a tumor suppressor gene. Oncol Lett.

[30] Glisovic T, Bachorik JL, Yong J, Dreyfuss G (2008). RNA-binding proteins and post-transcriptional gene regulation. FEBS Lett.

[31] Wagner EJ, Carpenter PB (2012). Understanding the language of Lys36 methylation at histone H3. Nat Rev Mol Cell Biol.

[32] Li J, Duns G, Westers H, Sijmons R, van den Berg A, Kok K (2016). SETD2: an epigenetic modifier with tumor suppressor functionality. Oncotarget.

[33] Wu Q, Lian JB, Stein JL, Stein GS, Nickerson JA, Imbalzano AN (2017). The BRG1 ATPase of human SWI/SNF chromatin remodeling enzymes as a driver of cancer. Epigenomics.

[34] Skoulidis F, Heymach JV (2019). Co-occurring genomic alterations in non-small-cell lung cancer biology and therapy. Nat Rev Cancer.

[35] McGranahan N, Swanton C (2015). Biological and therapeutic impact of intratumor heterogeneity in cancer evolution. Cancer Cell.

[36] Gerlinger M, Rowan AJ, Horswell S, Math M, Larkin J, Endesfelder D, Gronroos E, Martinez P, Matthews N, Stewart A (2012). Intratumor heterogeneity and branched evolution revealed by multiregion sequencing. N Engl J Med.

