# Supplementary material for: Mutation profile of non-small cell lung cancer revealed by next generation sequencing
Source: Respir Res. 2021 Jan 6;22:3. doi: 10.1186/s12931-020-01608-5 (PMC7789556; doi:10.1186/s12931-020-01608-5)
Supplement: Supplementary file 2 — Additional file 2: Figure S1. Kaplan–Meier survival curve of patients with EGFR, KRAS, TP53, and CTNNB1 mutations. Figure S2. Kaplan–Meier survival curve of 18 patients with EGFR mutation TP53 wild-type versus 8 patients with EGFR/TP53 mutations. Figure S3. Kaplan–Meier survival curve of 8 patients with EGFR mutation TP53 wild-type versus 3 patients with EGFR/TP53 mutations after EGFR-TKI treatment. [file 12931_2020_1608_MOESM2_ESM.docx]

**Figure S1.** Kaplan-Meier survival curve of patients with *EGFR*, *KRAS*, *TP53*,

and *CTNNB1* mutations.


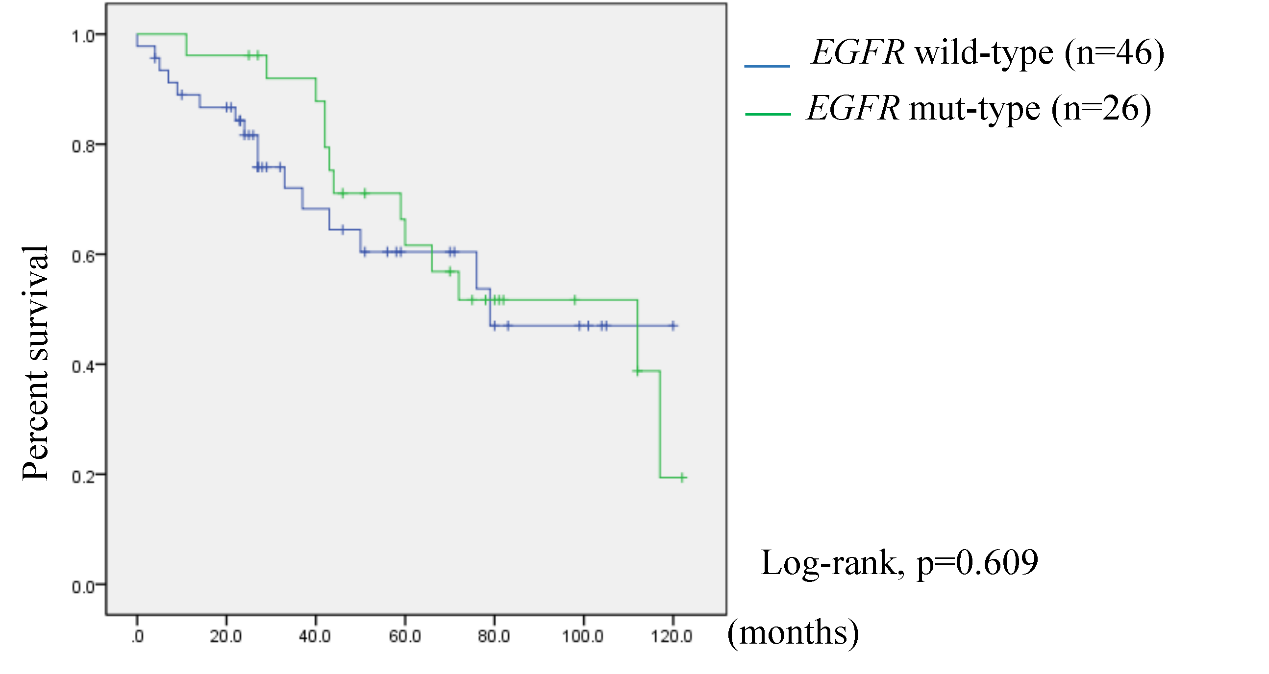


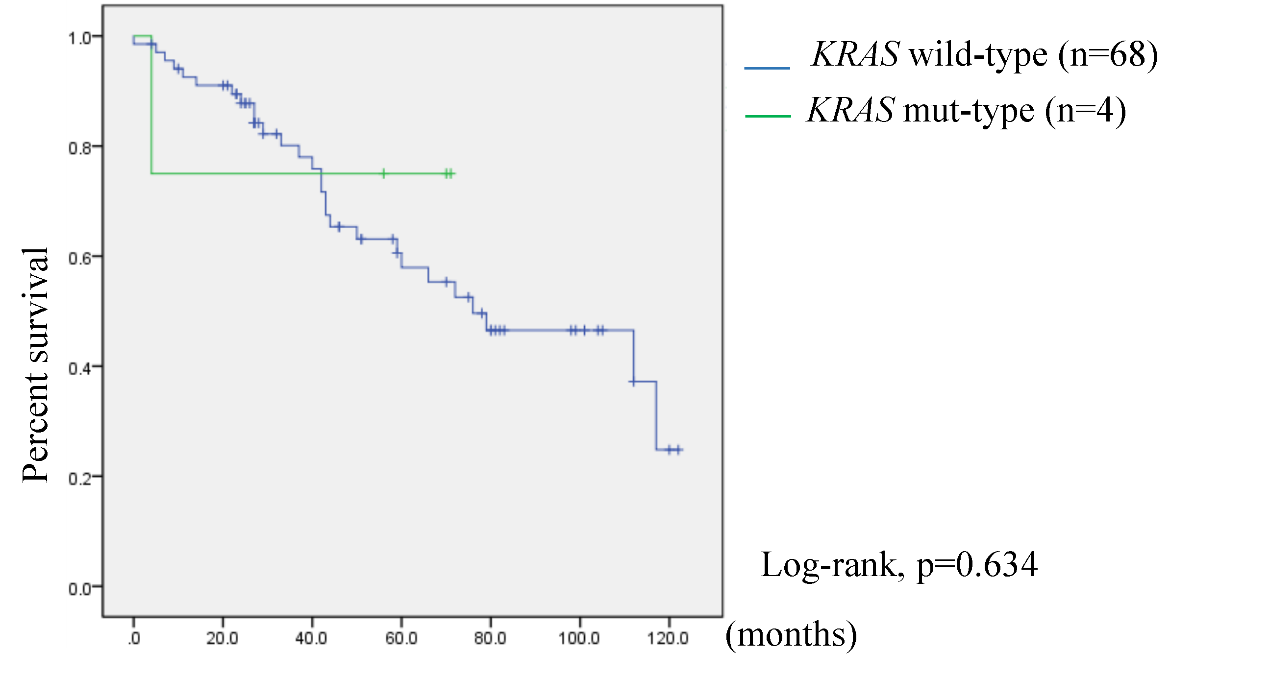


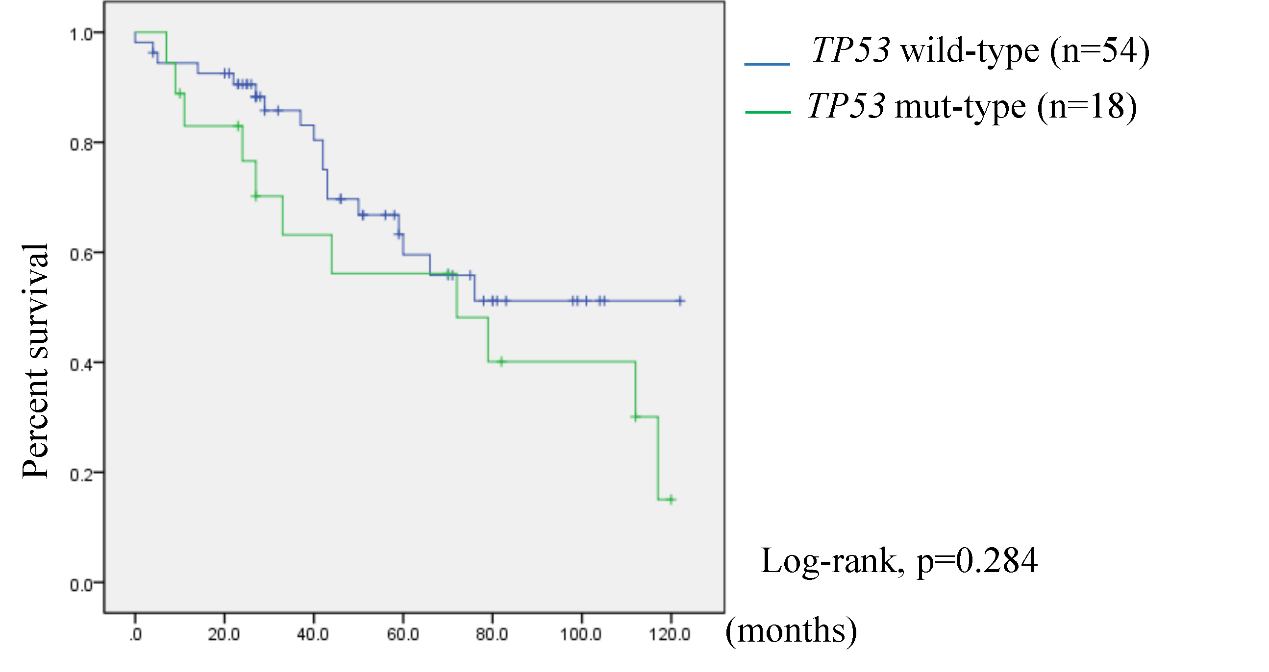


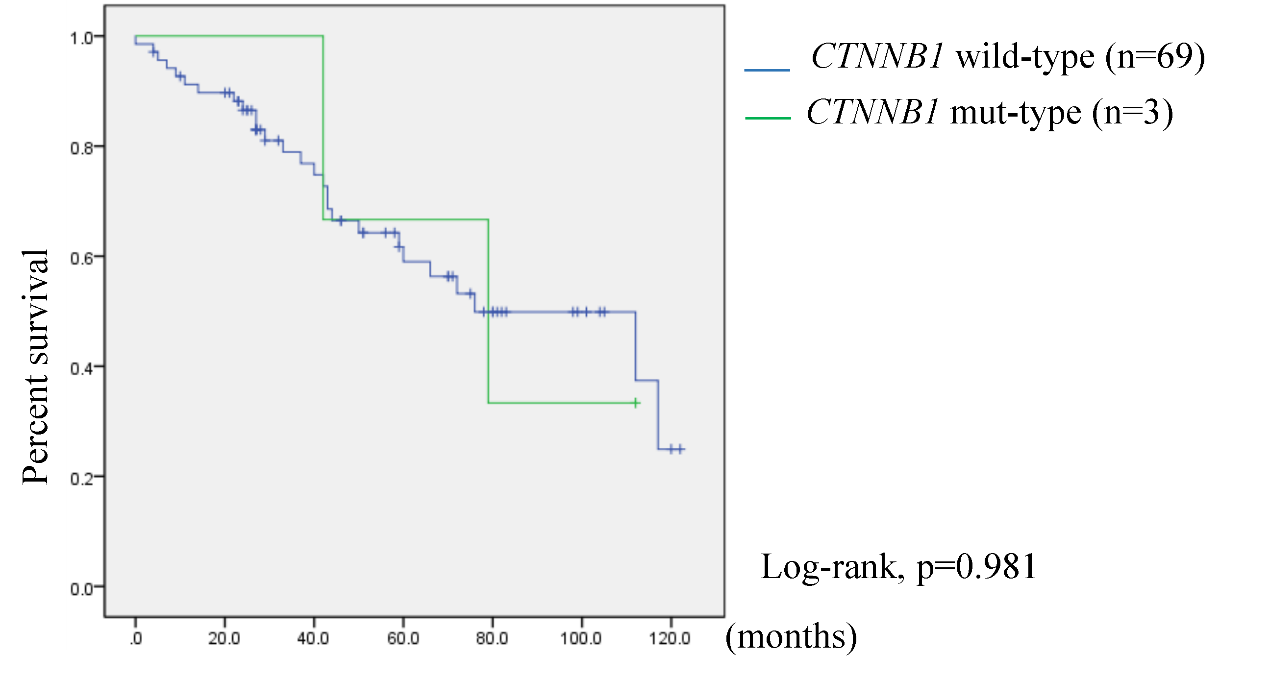


**Figure S2.** Kaplan-Meier survival curve of 18 patients with *EGFR* mutation *TP53* wild-type versus 8 patients with *EGFR/TP53* mutations.

**
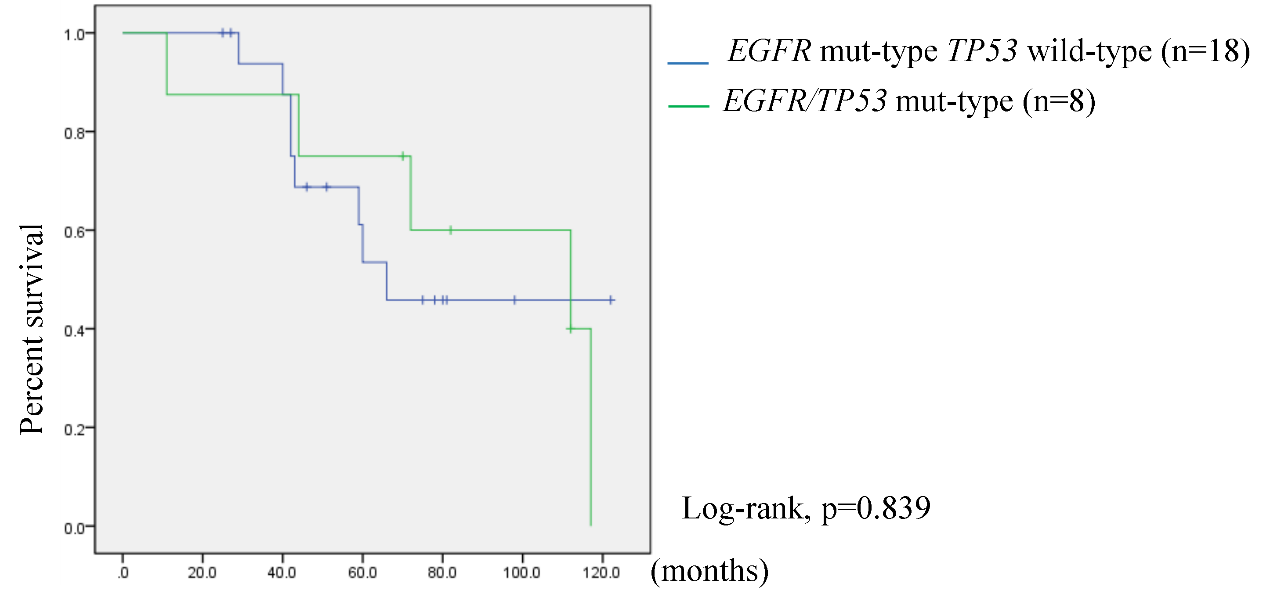
**

**Figure S3:** Kaplan-Meier survival curve of 8 patients with *EGFR* mutation *TP53* wild-type versus 3 patients with *EGFR/TP53* mutations after EGFR-TKI treatment.

**
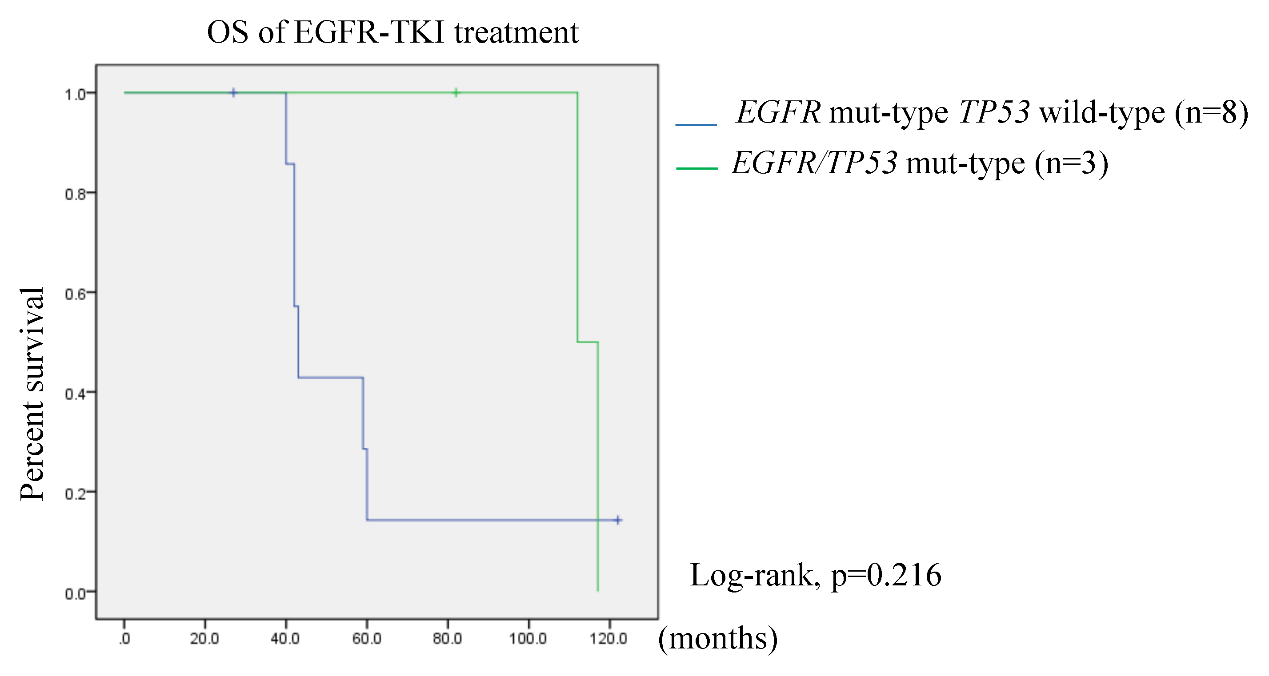
**
